# Intergenerational Integration in Community Building to Improve the Mental Health of Residents—A Case Study of Public Space

**DOI:** 10.3390/bs13040292

**Published:** 2023-03-29

**Authors:** Jianbin Wu, Kin Wai Michael Siu, Linghao Zhang

**Affiliations:** 1 School of Design, Jiangnan University, Wuxi 214000, China; 2 School of Design, The Hong Kong Polytechnic University, Hong Kong, China

**Keywords:** community building, community mental health, community psychology, design strategy, intergenerational integration, public issues, social well-being

## Abstract

This study defined intergenerational integration in communities at a theoretical level and verified whether a series of measures could facilitate negotiation and communication between community residents and other stakeholders to generate a positive and healthy community environment and gradually improve intergroup relations. Specifically, we applied community psychology and used Hongqiao New Village in Shanghai, China, as a research site to explore intergenerational conflict in public community spaces. The research was divided into two stages: an input stage and an output stage. In the input stage, participatory research and tea parties were used to deeply explore residents’ public space requirements. In the output stage, we tested the validity of the theory by using the Intergenerational Attitude Scale to investigate whether the intergenerational relationships were changed by the co-creation intervention. The results showed that the intervention caused a decrease in the incidence of conflict between residents using the square and caused some children to join the older groups in their activities. We thus propose a theoretical system model of intergenerational integration strategies that incorporates elements of integration, disagreement, and synergy in intergenerational interactions. Overall, this paper provides new ideas for building a community environment that supports mental health and improves intergenerational relationships and social well-being.

## 1. Introduction

Given aging populations and the implementation of social policies worldwide, there is a need to increase the responsiveness of older people to an active aging environment, which means enhancing the vitality of the older population and supporting the active participation of older groups in socio-economic activities and public life [1]. Scholars have therefore begun to examine the aging context from an intergenerational perspective to help different generations work together to build a more inclusive “aging-friendly society” [2]. 

According to current research, intergenerational relationships include more than the two opposing trends of intergenerational conflict and integration. Intergenerational conflict arises whenever the interests or ideals of one generation collide openly with those of another. This issue is a series of alternating “violations” and the resulting interaction of “disconnection” [3,4]. Intergenerational conflict is also present in urban public spaces. The influence of age-related stereotypes is particularly evident in spaces created for children and youth, which are often seen as a conflict between older and younger users of public space, positioning the young as a source of fear and worry for the elderly [5,6]. However, intergenerational conflict also has an irreplaceable positive effect: it can contribute to the growth of both parties and improve mental health by deepening positive intergenerational relationships [7,8].

Intergenerational integration is being widely used and expanded to address the aging problem. Intergenerational integration is characterized by mutual interaction, and its durability relies on organic integration between older groups and other generations. Thus, developing intergenerational practices can enhance intergenerational closeness, understanding, and communication and foster a commitment to reciprocity and solidarity [9]. In addition, accommodation theory strategies in the field of communication focus on integration and divergence, with integration manifesting as collectivism and emphasizing social community, and divergence focusing on the individuality of the personalities themselves. Although the two focus on different points, they can both be effective theoretical strategies to promote intergenerational relationships [10,11]. During the COVID-19 pandemic, intergenerational issues received increasing attention, and the concepts of intergenerational integration and responsibility became prominent.

A community is an orderly social group comprising multiple elements, such as geography, spirituality, blood, and labor relations [12]. The renowned German sociologist Ferdinand Tønnes noted that “community” and “society” are two structural elements essential to the organization of any group, where the conscious association of people is the internal latent aspect of the group, i.e., its community, while the external dimension is the promotion of association through institutions, i.e., society [13].

The motivation for creating a community arises from two main sources. The first source is external and is related to the political form, economic development, and social welfare of the community and society at large; the second source is internal and represents people’s “sense of presence” in their inherent environmental, ideological, and sociocultural identity. The theory of individual interaction in social interaction states that interaction between individuals involves inter-individual attraction, nonverbal communication, stereotypes, and inter-individual space [14,15,16]. It also predicts that future intergenerational integration of communities will be driven by the construction of intergenerational relationships, cultural shaping, the cultivation of community consciousness, and other intrinsic motivations. Such intergenerational integration will involve various methods and mechanisms requiring in-depth research and exploration, which will be of academic value and practical significance.

Furthermore, community psychology is an effective, action-oriented field with theories that emphasize improving the quality of life of individuals, communities, and societies. Community psychology is committed to group collaboration to create social and community change that benefits individual members of the community and the collective [17,18,19]. At the same time, this definition masks potential tensions within the discipline regarding the role of broader social and political power in shaping communities [20].

Although it is possible to explain the importance of intergenerational integration in community development from a theoretical perspective, there is a lack of systematic research and models on how to implement it and what ways and means there are to intervene to promote intergenerational relationships. Therefore, this topic focuses on two main questions: 

(1) What are the core elements that promote intergenerational integration among communities?

(2) In the context of community building, can we promote intergenerational awareness and build a positive community mental health environment by engaging residents in collaborative interactions?

(3) What are the specific strategies for implementing integration and divergence in intergenerational relations?

These questions deserve further discussion and exploration. In the current environment, the COVID-19 pandemic has changed people’s lifestyles and affected the population’s mental health. The loss of livelihood and loved ones, social rejection, and intergenerational tension are even more pronounced in times of crisis. There is also an increased risk of mental health problems, including depression and anxiety, due to hindered interaction and distancing between people [21]. Therefore, in the face of current complex and diverse situations, more proactive measures should be taken to meet the key challenges and achieve sustainable community development.

This study explored the possibility of using communication platforms and participatory activities to enable community residents to negotiate, cooperate, and articulate their real needs when public problems arise in a community. We designed a series of activities as group-based problem solving can improve relations between groups. We tried to explore the possibility of creating a positive and healthy environmental atmosphere by emphasizing the group’s self-worth through intergenerational solidarity, weakening social class relations and reducing the psychological stress of the local group.

## 2. Literature Review

### 2.1. Community Building and Mental Health

Community building is a complex and systematic innovation process [22]. The prototype of community creation originated in the early 20th century in several community-building movements in the United Kingdom, United States, and France. The current concept of community creation is considered to have an Eastern flavor and formally emerged in Japan in the 1970s. Community creation in Japan has since progressed through three main stages. The first stage focused on solving basic problems, such as the deterioration of the living environment. The second stage (1980s) encouraged citizens to participate in urban planning; the conservation of cities, historical districts, and communities; the improvement of the living environment of communities; the preservation of local culture; and the establishment of local government. The third stage occurred after the 1990s, when the Japanese government revised laws and regulations, including the Land Law and the Urban Planning Law [23]. At this stage, the government, nonprofit organizations, and citizens gradually started working together to solve problems. Citizens started becoming important players in community development, the numbers of volunteers and social organizations increased rapidly, and relevant laws and regulations were gradually expanded and improved. This eventually led to the creation of a community that combined government control with public participation [24,25,26,27].

Furthermore, community building is regarded as a dynamic process whereby residents in a certain geographical area establish strong social ties through interaction and communication. The core of community creation is public participation, which emphasizes public spontaneity, and community creation can be used as a concept and methodology to guide community residents to participate in community building and management on their own [28,29]. The community creation context also allows groups to improve their community environment by discussing and dealing with community issues together, thereby enhancing residents’ sense of community identity, promoting strong social ties between residents, and increasing community-to-community ties to form a large community and promote healthy and sustainable social development [30]. That is, residents collaborate to discuss community issues and solve community problems by combining their collective wisdom to contribute to the sustainable development of the community.

The primary foci of community mental health services are treatment, palliation, relapse avoidance, primary prevention, and health promotion. Common to these is the role of cultural elements (e.g., cross-cultural elements) in facilitating the formation of a community framework. Consequently, concepts such as community mental health care and community mental health management have emerged [31,32]. In addition, the overall environment of the community, along with its subjective group interactions, can also influence the mental health of residents [33,34]. This effect is called the “neighborhood effect”, meaning that a community’s environment directly or indirectly influences the way local residents think and behave, thus affecting their health, behavior, and health status [35]. This suggests that diverse and rich community co-creation activities can be effective in bringing residents together and alleviating negative emotions by maintaining a positive and healthy community.

The community is also trying various research methods, such as using art to alleviate the psychological impact on the body, as in the case of Boro-Hanna, to effectively manage various mental health and emotional problems [36]. People such as Heather-Gredley organize community art choirs to promote public cohesion among community residents, enhancing their confidence, abilities, happiness, and interpersonal skills [37]. It also shows that urban communities, community safety, and neighborhood interaction play an important role in residents’ mental health. In a follow-up study, we aim to strengthen the dissemination of mental health information, focus on social teams and group activities to enhance residents’ mental health status, and make it easier for residents to obtain material and emotional support through community network interactions [38].

### 2.2. Community Intergenerational Integration Model

Vincent proposed intergenerational integration and intergenerational solidarity as two dimensions of the ideal intergenerational relationship. Intergenerational integration focuses on overall intergenerational harmony, while intergenerational solidarity focuses on the level of cohesion inherent to intergenerational relationships [39].

At the level of intergenerational relations, there are two distinct but related types of intergenerational integration. The first type involves breaking down age barriers, so that age cannot be used to determine the positions or roles people can or cannot hold. The second type is “cross-generational interaction”, in which groups of people of different ages work together, for example, at work, school, or play. These two types of age integration are related because neither type is accompanied by the other. For example, when a work organization welcomes many people of different ages, it is likely to have people of different ages working together. However, they remain distinct types of age integration, as organizations or societies that feature one type of age integration do not necessarily feature the other.

**Figure 1:**
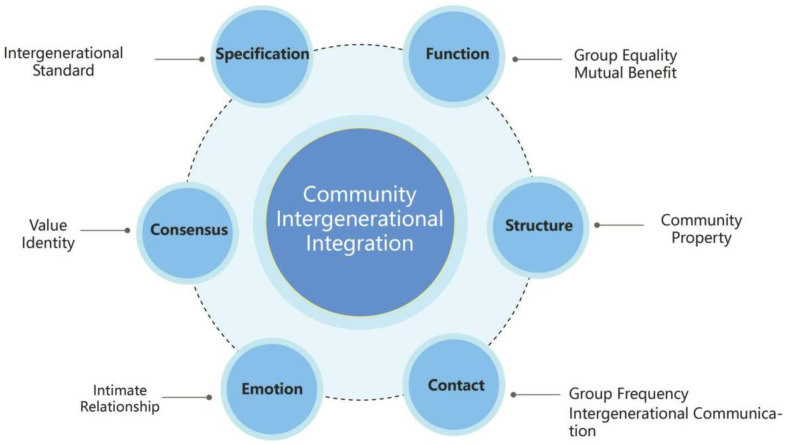
Community intergenerational integration model. The functional element emphasizes the equality of rights of intergenerational groups; the structural element includes community development, attributes, and group structure; the linkage element refers to the frequency of group communication and participation; the emotional element focuses on intimacy; the consensus element focuses on the sense of equality; and the normative element represents mechanisms and guidelines.

Furthermore, intergenerational integration theory holds that behavior is a key component in the promotion of intergenerational integration. In addition, intergenerational integration behavior is closely related to the attributes of function, emotion, connection, norms, and organization, all of which are part of intergenerational integration theory [40]. Figure 1 outlines our definition of an intergenerational model based on intergenerational theory in a community context.

The establishment of a model of intergenerational integration in community contexts can serve as a theoretical foundation for researchers to conduct targeted research activities specifically in terms of function, structure, contact, emotion, consensus, and specification dimensions. Therefore, intergenerational integration behavior is an important pathway to promote intergenerational integration. In particular, diverse intergenerational interactions enable different age groups to benefit and gain insights through socialization, learning, and cognitive processes [41]. For example, younger groups can help older groups understand new ways of thinking, and older groups can pass on their wisdom and experience to younger groups, thus gradually alleviating the stereotypes younger groups hold about older people, and helping older groups perceive themselves as valuable to society, thereby reducing their feelings of social isolation.

## 3. Methods

We used a community in Shanghai, China, as the project platform to systematically study intergenerational conflict and contradictions in public spaces. We intervened in the project process by using methods based on the concepts of “integration” and “divergence” to test whether co-creation and communication are conducive to intergenerational relationships.

### 3.1. Program Background

The Shanghai Hongqiao Airport New Village is a family area with civil aviation associations, as all of its initial residents were civil aviation soldiers. Over time, the village population has grown to more than 7000, and its proximity to the civil aviation hub means that many of its new residents are also connected to the civil aviation industry. As a result, the Shanghai social enterprise organization Big Fish Creation created China’s first aviation-culture-themed community museum in the village based on its cultural heritage.

The project started by focusing on conflicts that occurred in the square in front of the museum. Specifically, during the peak usage time in the square, while women were square dancing, children were playing, and parents and elderly people were talking, the disorderly trajectory of children moving in and out of the square-dance line led to frequent conflicts and friction between the square dancers, the children, and their parents. The local neighborhood council indicated that before the team became involved in the study, the public plaza was often the scene of arguments, and that they even sought police assistance to regulate the relationship. Over time, these conflicts developed into stereotyping of the various groups. Thus, these conflicts harmed neighborhood relations and the friendly and sustainable development of the community. Therefore, this project, in collaboration with the Shanghai social organization Big Fish Creation, analyzed the relationship between the community groups and the square in an effort to identify the causes of intergenerational conflict in the community and find a way to resolve it.

### 3.2. Program Plan

Based on the community intergenerational integration model, we developed a detailed project plan, which was divided into an input stage and an output stage. In the input stage, a participatory research approach was used to address the real needs of the people; in the output stage, a collaborative co-creation workshop was conducted to encourage people to discuss and solve the conflict problem according to their current needs [42]. The implementation plan is shown in Table 1.

### 3.3. Program Process

#### 3.3.1. Community Participatory Research

Based on information collected in the early stages, the research team found that weekends were the peak time for community group activities. The team, therefore, introduced the concept of community days and organized multiple activities to encourage groups to interact. In the community research phase, the team did not use a one-on-one semi-structured interview, as it involves designated roles of interviewer and interviewee; instead, the team used a role-free research method that allowed residents to freely choose to participate in discussing issues that interested them and encouraged them to articulate their real needs in a game format. Community participatory research as a research method and tool not only addresses inertia in the field of community mental health but is also an attitude that builds positive relationships of mutual respect and sharing with the community and promotes deeper collaboration between intergenerational groups [43,44]. This research process lasted 5 days.

**Figure 2:**
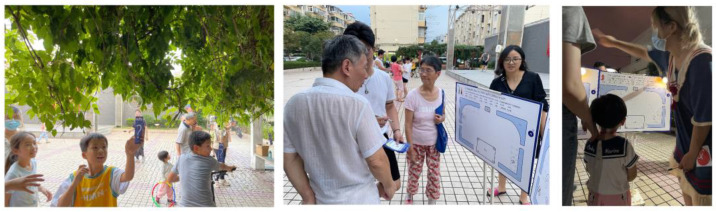
Community participatory research on-site activity. This activity was used to ask the crowd what activities they were actively participating in in the square space.

The research team used “Community Truth or Dare” as the activity theme and posed questions about the square related to the dimensions of the space, intergenerational relationships, and unrestrained thinking (Figure 2). The research questions were presented in the form of cards that addressed the use and perception of the public square by community members and the intergenerational distance between community members. The intergenerational content was adapted from the NCSR and GHQ-12 questions, which combined community participation and psychological well-being in neighborhoods and focused on the frequency and extent of local residents’ participation in social and public activities and discussions about community issues, for example, “Does interacting with my neighbors influence my decisions about my neighbors” and “There are many places in my community where I can stop and talk with others” [45,46,47,48]. The questions on the cards were combined with pictures and patterns of the scenes and placed on the actual site, which increased the readability of the cards’ content and enhanced people’s sense of involvement.

**Figure 3:**
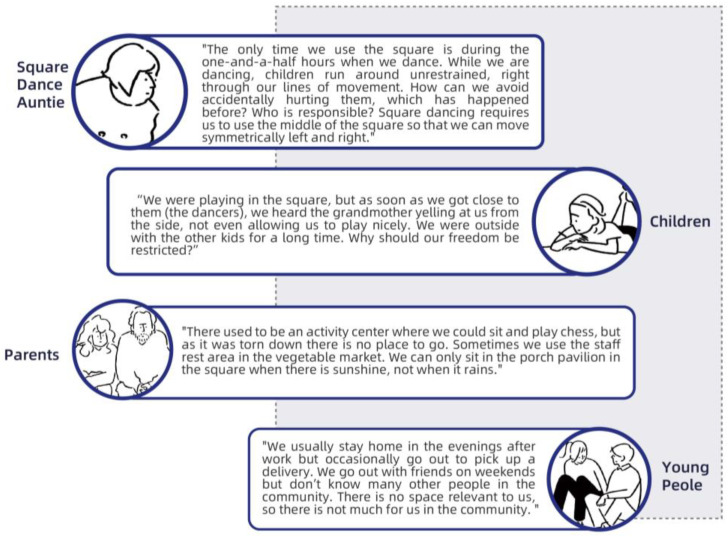
Community residents’ views.

During the participatory research process, the residents articulated many real self-needs through the interactive methods, i.e., games and question answering. Analysis of these needs revealed that the conflict between the groups was mainly due to their belief that the square was for public use and thus they had the right to use it. Through community participatory research, we learned that residents of all ages have many needs for public spaces, such as recreation, socialization, and rest. Regarding intergenerational relationships, the research results revealed that conflicts between children and older groups are more prominent, mainly focusing on square activities. To further understand the specific reasons for the conflicts, we conducted user interviews with community residents of all ages after the participatory research, combined with research information from community residents. The research showed that, although there was nothing wrong with this starting point for each group, the lack of empathy the groups showed for each other—as demonstrated by various reasons group members gave for opposing others’ interests—made the conflict serious. Figure 3 shows excerpts of the views of some residents.

#### 3.3.2. Establishing a Common Vision

Based on the information collected from local residents, it is clear that the intensification of group conflicts is driven by public interest and the lack of appropriate communication channels, which leads to the intensification of conflicts and even affects physical and mental health [49]. Therefore, based on the research aims, the research team summarized the list of needs of the community and ranked them in order of importance and specific insights. To gain widespread acceptance from the community residents and verify the reasonableness and realism of the needs, the team, together with the community council, invited local residents of all ages to a “tea party” event. Subsequently, given the diverse needs of the residents, the team held several more tea party events, and the community responded positively, elaborating on their needs and making suggestions after viewing the list of needs. This tea party format helped to improve the relationships between residents and the community, increased residents’ understanding of the community’s concern for them, and led to residents gradually developing a sense of ownership.

#### 3.3.3. Community Co-Creation Workshop

Through the tea party events, the team not only established a consensus with the community residents but also confirmed the authenticity of the team’s needs list. Therefore, the focus of the output phase was to generate an effective solution and gain the approval of the residents.

**Figure 4:**
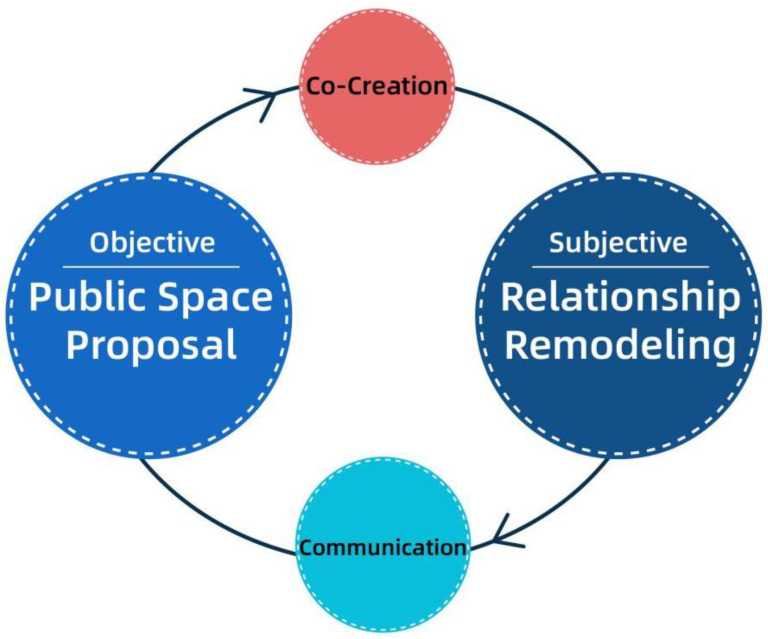
Core framework of co-creation workshop.

As the research results showed that some of the relationships between the residents of the community had soured due to problems with the use of public space, the team organized a collaborative workshop and invited residents, especially those involved in conflicts, to participate. The purpose of the workshop was to create a collaborative approach to identify collective solutions to the conflict and build a platform for residents to communicate with each other about the conflict [50]. See Figure 4.

The design of the community workshops was discussed at length. Based on the elements of intergenerational integration and co-creation, it was decided that a community workshop should be easy for a general group with strong internal connections to participate in, as this would encourage participation. Therefore, for the overall process, the team used “impressions of community squares” and “designing the ideal community square” as the topics, and the workshop structure was set as follows: icebreaker, community impressions, square co-creation, evaluation, group photo.

In the co-creation workshops, the residents proposed many creative design ideas, and the concept of “everyone is a designer” was evident in the overall design process. For example, a 10-year-old child wanted to transform the community lotus pond into a swimming pool and viewing pond, thereby allowing children to swim and older people at the pavilion to enjoy the view; another child’s parents envisioned transforming the viewing path into a running and bicycle path to facilitate children’s activities [51].

#### 3.3.4. Concept Iteration and Evaluation

Although many effective solutions were proposed at the co-creation workshop, the output from the workshop represented the suggestions of only some groups, whereas a community design should meet the needs of a wide range of groups. Therefore, the research team conducted a new round of information gathering to lay the foundation for concept iteration.

First, the team created “Plaza Village Message Boards”, on which residents could freely express their suggestions for different venues in the community. These message boards were posted on a community museum display window, in a community restaurant, in a logistics center, and in other areas with high traffic flow, so that residents could easily access them. Second, a peer-to-peer approach was used to provide residents with existing design solutions and ideas on how best to use them. Finally, the research team worked with various community stakeholders and resident representatives to discuss and conclude the project. These proof-of-concept activities continued for 10 days.

**Figure 5:**
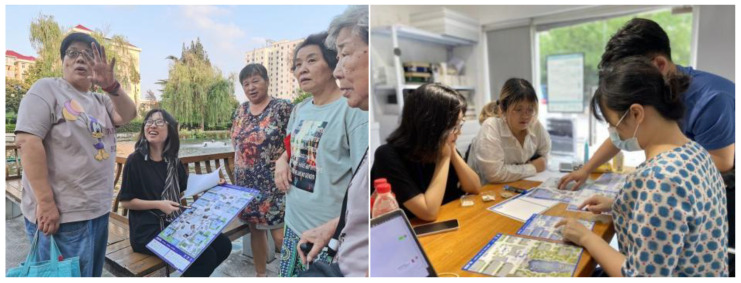
Active participation of community residents in the discussion. We iterated on the community space approach by giving the co-created community map to more local residents for suggestions.

In this way, the needs that had been identified and the solutions that had emerged from public co-creation were improved and clarified through iteration. In addition to the rational organization of the research team, community leaders also played an important role, as they “built bridges” between the team and the community and gathered their friends and family to complete the iterative process while offering valuable suggestions themselves. In addition, as older people were relatively less involved than other groups, the team sent young college students to the places where older people gathered in the community to conduct “one-on-one” inquiries and elaborate on the original proposal, to ensure that the older people effectively understood the purpose of the activity. As a result, even older people who were initially reluctant to express their opinions gradually became willing to express themselves and invited other older people to join in the discussion. See Figure 5.

#### 3.3.5. Community Exhibition

With community participatory research, user interviews, tea party focus groups, and co-creation workshops, the team gained a deeper understanding of the needs and visions of the local naming of public spaces. From an objective point of view, the main source of conflict among residents is the lack of public space and the poorly stocked public facilities, which reduce the length of time a section of space can be used, thus causing overcrowding, which leads to a series of conflicts and exacerbates intergenerational tensions. On the other hand, the plaza is not only a space for children to play with their families and for the elderly to rest but also a cultural symbol rooted in the hearts of generations of residents. Instead, the research team redefined the community plaza by taking into account the needs and suggestions of the residents.

In terms of basic needs, community plazas can be considered “supply stations”, providing a variety of conveniences for community residents, and they should have restrooms and beverage vending machines; in terms of safety and security, they should have strong protective measures, backrests for seats, and high fences; social needs should be met by the cultivation of friendly relationships among all age groups through activities such as community festivals, tea parties, and lectures. “De-labeling” focuses on the respective needs of the group, distinguishing between public and exclusive. Finally, the demand for self-actualization focuses on establishing a “window” for cultural connotation and spiritual display, strengthening communal cultural symbols, and promoting community participation and co-building.

**Figure 6:**
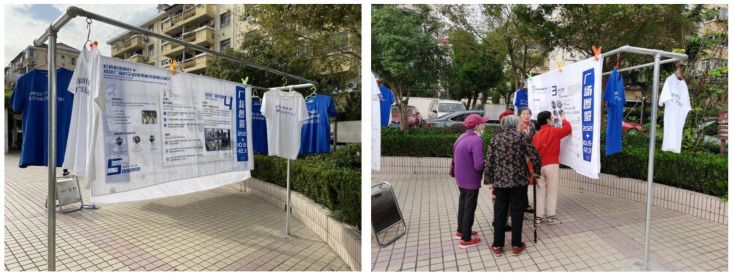
Community illustration exhibition. The Community Illustrated exhibition focuses on the real and diverse needs of residents in public spaces and the team’s specific research. Promoting good neighborly relations between groups through information displays.

To help residents understand each other’s needs and opinions about the square space, we held the exhibition “Community Illustrated” in the community square, using the form of quilts to show the residents the research process, the redefinition of the square, and the co-creation of the group. Through the exhibition, we sought to promote intergenerational understanding and unite groups (see Figure 6).

### 3.4. Results

#### 3.4.1. Qualitative Analysis

**Figure 7:**
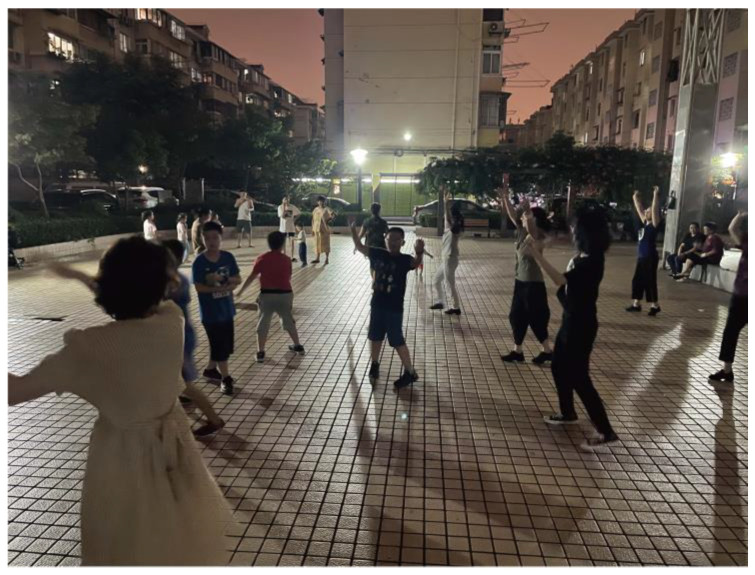
Older adults and children interacting in the square.

The research team followed a co-creation theoretical approach from the initial immersion research to the subsequent implementation of the residents’ co-creation design activities. The project lasted nearly 2 months, and through the efforts of the team and their helpers, the relationships between the residents underwent a subtle change. To verify the concrete effect of the design intervention, we conducted regular participatory observations in the community square during the project’s process [52]. At the beginning of the project’s intervention, there were many incidents of conflict. We helped to coordinate and understood the causes of the conflict and invited the residents involved in the conflict to join our design process. As the project continued, intergenerational relationships improved through collaboration and co-creation as residents developed empathy and an understanding of each other. Therefore, in the later stages of the project, our observations found that the number of angry interactions in the square between residents decreased, and children could even be seen dancing with older people (see Figure 7), indicating that the application of co-creation theory during the design process improved the relationships between residents. After the project ended, the research team continued to follow the dynamics of the community, and the number of conflicts did not rebound.

In addition, the team interviewed the residents after each activity to determine what they had experienced and felt during the activity and obtain their suggestions on how to improve the activity. This revealed that the residents generally found that the workshops helped them to give suggestions and to gain insights into the real needs of other groups through dynamic discussion and collaboration, which generated empathy and reduced the chance of conflict. Table 2 presents some of the real interviews conducted with the community residents.

#### 3.4.2. Quantitative Analysis

To understand the perceptions and attitudes of different groups, the team used the Intergenerational Attitude Semantic Scale (Polizzi) to examine the generational shift of 30 residents before and after participation in the activities [53]. The 30 residents took part in the participatory research, co-creation, and iterative processes, and ranged in age from 10 to 65 years, comprising 10 residents from each of the three groups: adolescents (10–18 years), young and middle-aged adults (18–50 years), and older people (50–65 years). The list of intergenerational semantics is shown in Table 3.

**Figure 8:**
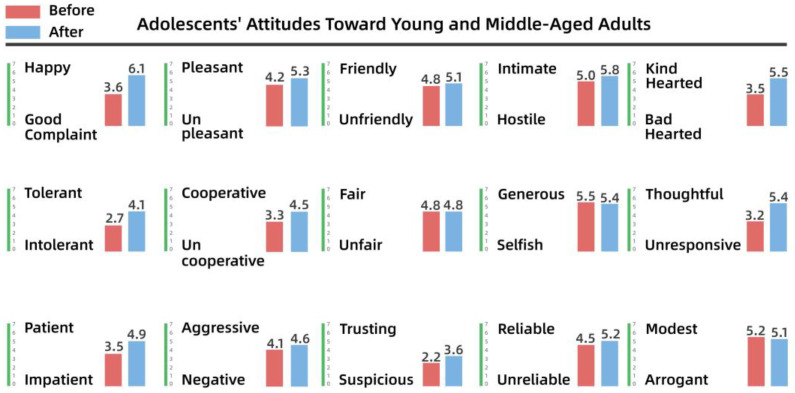
Adolescents’ attitudes toward young and middle-aged adults.

**Figure 9:**
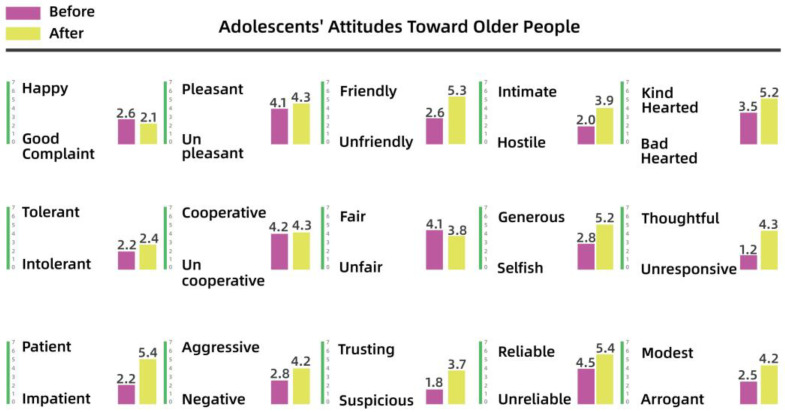
Adolescents’ attitudes toward older people.

**Figure 10:**
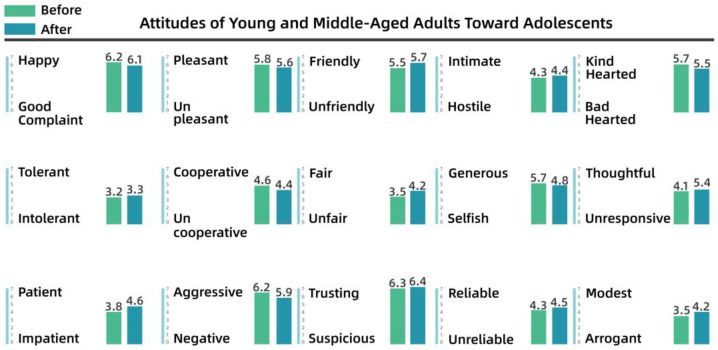
Attitudes of young and middle-aged adults toward children.

**Figure 11:**
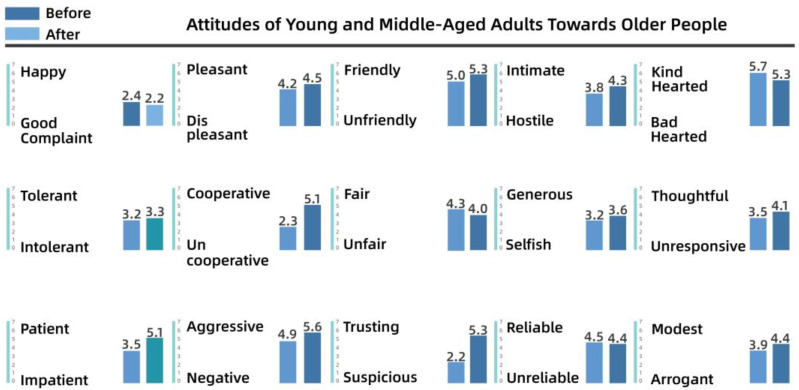
Attitudes of young and middle-aged adults toward older people.

**Figure 12:**
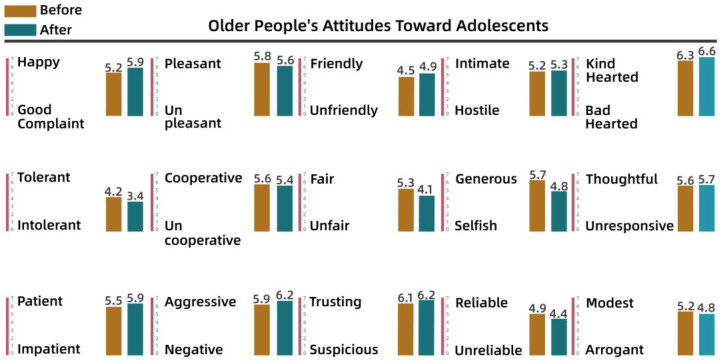
Older people’s attitudes toward adolescents.

**Figure 13:**
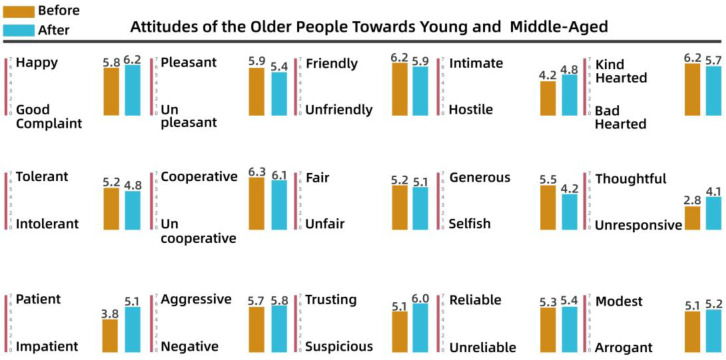
Older people’s attitudes toward young and middle-aged adults.

Pre- and post-test numerical analyses indicated that a shift in attitudes had occurred among the residents during the campaign and exhibited a positive trend overall. The analysis is shown from Figure 8, Figure 9, Figure 10, Figure 11, Figure 12 and Figure 13.

(1) Semantic scale results for adolescents’ attitudes toward adults

It is clear from Figure 8 and Figure 9 that the children’s attitudes toward both adult groups changed after the intervention, and that this change was generally in a positive direction. However, the change in children’s impressions of young and middle-aged adults was small, ranging from 0.2 to 2 percentage points for each variable, while the change in their impressions of older people was larger, especially for “friendly–unfriendly”, “patient–impatient”, and “generous–selfish”, which improved by 2 to 3 percentage points. This also indicates that the children’s stereotypes of other groups had gradually changed through participation in dynamic activities over time.

(2) Semantic scale results for young and middle-aged adults’ attitudes toward adolescents and older people

Figure 10 shows that there was little change in the attitudes of young and middle-aged adults toward children after the intervention, although they were generally more positive, with higher weights for “happy”, “negative”, “generous”, and “trusting”. As shown in Figure 11, before the intervention, young and middle-aged adults perceived the older group as more “complaining”, “uncooperative”, and “intolerant”, whereas after the intervention, they perceived the older group as more “cooperative” and “trusting”. After the intervention, apart from the substantial increase in “cooperative” and “trusting”, other variables increased by only a small magnitude. The team determined that this lack of a significant effect after the intervention indicated that the cognitive system of the young and middle-aged adults was relatively stable.

(3) Semantic scale results for older people’s attitudes toward younger age groups, young and middle-aged people

Figure 12 and Figure 13 show that although some older people were in conflict with other groups over public space issues, their overall attitudes were more positive, with low variability before and after the intervention. This led to further consideration of whether older adults had positive perceptions of others. In fact, some residents reported that some older adults showed negative emotions and attitudes toward other groups. This disparity suggested an urgent need to provide a communication platform.

Therefore, based on the quantitative intergenerational data, it was decided that a collaborative interactive intervention in the overall project process could facilitate and change the attitudes of the groups, especially those of children and older people, toward the community. In addition, although there were no significant differences in intergenerational attitudes among middle-aged people, their willingness to communicate and interact with other groups in the community increased, as seen in the qualitative interviews after the design intervention. This suggests that adopting a reasonable and effective approach to intervention in community creation is conducive to improving intergenerational relationships and mitigating intergenerational conflicts.

## 4. Discussion

Based on our practical project summary, we found that when devising public events for all groups, appropriate methods were required to collect information about people’s needs, give them the opportunity to communicate with each other, reach a consensus, and ease intergroup relations. The results of the qualitative research and the quantitative analysis indicated relative success, including fewer incidents of conflict, less intergenerational distance between community residents, and a more pronounced shift in intergenerational attitudes. However, there are still many shortcomings and reflections that arise with each phase. We re-evaluated the methodologies involved and present below the main issues in implementing each methodology.

### 4.1. Project Phase Review and Reflection

Community participatory research. (1) The universality of the activity format is weak, and the participants of the weekend community day were mainly children and elderly groups, while the participation of other groups was relatively low. The reason for this is that young and middle-aged people do not have a high demand for public space in the community, the community is relatively closed, and the team has not promoted extensive publicity. (2) Some of the questionnaires are obscure and difficult to understand, such as mental health and intergenerational relationships, making them incomprehensible to some residents and requiring special explanations from the researcher to complete. Therefore, activities for all ages should be universal in theme and content to reduce group frustration and trial and error. (3) Intergenerational interaction is low. There is a lack of activities that focus on public space plazas and are discussed together through intergenerational discussions.

Shared-vision tea party. The purpose of the tea party is to rebuild the residents’ emotional bond and build a bridge between them. It is an opportunity for groups to discuss issues that need to be addressed in the community and to present their needs and opinions. In practice, the tea party also encountered situations where young and middle-aged groups were less motivated to participate. The reason for this is that it is not easy for this group to find support (like-minded people) in the community, and the content and format of the topics are not relevant to the needs of young people.

Community co-creation workshops. The problems that emerged from the workshops were mainly focused on the pre-recruitment as well as the dynamic implementation process. (1) The number of older groups participating was low. After the study, it was found that, in addition to not receiving relevant information dissemination channels, the main reason for this was that the older groups did not consider themselves suitable for such activities and were worried about being left out [54]. (2) During these activities, the intensification of conflicts between groups could lead to some of the processes being blocked. (3) The children’s groups lacked team awareness, had poor execution during co-creation, and did not understand some of the process elements. Therefore, in a follow-up study, attention needs to be paid to improving the self-confidence of the older group and to having experienced facilitators and “game” rules that are consistent with the children’s thinking [55].

Community public space exhibition. The combination of the exhibition “Community Square Illustrated” and the community square space is innovative and effective in dissemination. However, there is still room for improvement, which could include putting the research results into phases and opening them to residents after the completion of a phase to help residents understand the team’s development from scratch, thus maintaining the dynamics and “presence” of the research. Contradictions and issues that arise in the community are developed into topic cards and discussed regularly. With the rapid development of society, the community’s achievements should be presented richly and diversely. By working together to address the commonality between online and non-online communities, we can re-establish emotional bonding, further emphasize the self-worth of the group, reduce residents’ stress, and enhance the sense of belonging and intergenerational cohesion of the community.

Faced with the problem of intergenerational conflict in the public square space, the team learned through design interventions that intergenerational collaboration and communication can promote intergenerational relationships, reduce intergenerational conflicts, and improve the psychological well-being of community residents.

### 4.2. Theoretical Model of Intergenerational Integration Accommodation Strategies

We then summarized the theoretical model of intergenerational communication strategies and proposed further in-depth methodological pathways based on collaboration, communication, and experience.

As mentioned before, communication theory emphasizes two strategies: “integration” and “divergence.” At the design level, the “integration” strategy needs to focus on convergence between generations, i.e., the relationship of equality between two groups that is generated by their inherent dynamics and contributes to their identity. Integration is not homophily; rather, it focuses on the ability of intergroup consciousness and cognition to enable groups to support each other and reach a degree of consensus. Divergent strategies, on the other hand, emphasize individual uniqueness, especially in the process of implementation, wherein age groups are seen as comprising independent and autonomous individuals rather than one-sided stereotypes based on prejudices (see Figure 13) [56].

In addition, by combining theoretical research and design practice, the team propose a “synergistic” strategy, i.e., a collaborative co-creative intervention for the construction of relationships that serves as an effective driver of integration and divergence. This is manifested by designers or professional teams who use their expertise and tools to coordinate, activate, and interact with participants to create meaning [57].

The authors have found that the relationship between “integration” and “disagreement” can be balanced by combining academic research and practical activities [58,59]. That is, when dealing with complex and sensitive situations, we can attempt to make the main conflicts “public” and provide a platform for stakeholders to communicate through debates, workshops, tea parties, and council chambers. By using the platform, participants are involved in a series of processes, such as elaborating needs, collaborating with each other, and learning about each other’s situation, which gradually improve their perceptions of each other via a dynamic experience of consciousness clashes and exchanges.

**Figure 14:**
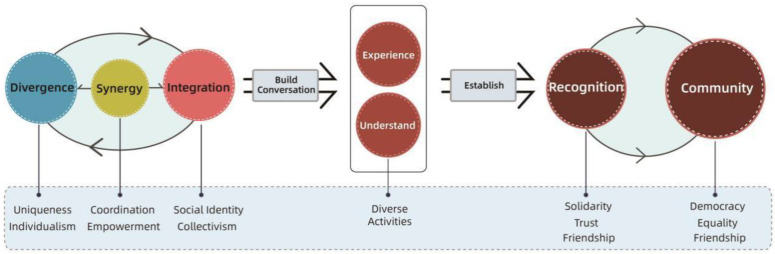
Theoretical system model of intergenerational integration accommodation strategies.

Therefore, the three strategies are organically integrated and intersect according to the multiplicity of contexts, and an “intergenerational dialogue” is formed through the rational use of accommodation strategies (integration, divergence, and synergy) in Figure 14. Dialogue here is not limited to verbal communication in its natural state; it also includes non-verbal communication, such as thematic seminars (workshops) and intergenerational synergistic activities [60]. The mental health of community residents needs to be enhanced in terms of participation, community well-being, and a sense of belonging [61,62]. Therefore, high-quality intergenerational dialogue facilitates communication between generations. After a certain duration and regular interaction, community members gain a new and better understanding of each other, which results in intergenerational friendship and the construction of intergenerational identity. In this process, collaborative interactions are gradually created, retranslated, and encoded into new imagery, eventually reaching intergenerational consensus and forming a social community with a certain sense of community belonging.

### 4.3. Strategies of Divergence and Integration

Based on the strategic theoretical model above, we further elaborate on how to enable community residents to balance the relationship between integration and disagreement when dealing with public situations in terms of collaboration, communication, culture, and visibility. In turn, we can alleviate group conflicts and create a healthy physical and psychological environment that helps solve problems at the system level.

#### 4.3.1. Emphasizing Intergenerational Collaborative and Competitive Relation Adjustments

The principle of moderation plays an important role in facilitating intergenerational communication. The premise of participant co-creation is based on participants being on an equal footing and free from prejudice. However, excessive convergence can limit creativity [63]. Janamia et al. noted the importance of competition in engaging in intercultural communication in design, where intense conflict could provide an opportunity for diversity in a co-creative environment, thereby stimulating and initiating co-creation between the various parties [64]. Therefore, when planning design activities, research teams should focus on the rational application of co-creation and competition in all phases of the process and control the points of emphasis between the processes. Older participants in particular should be encouraged to actively engage in dynamic activities and increase their interactions and discussions with participants belonging to other generations to enhance mutual bonding [65].

Moreover, as mentioned above, integration and divergence are not limited to confrontation and can be effective adjustment strategies. Researchers may therefore consider using a combination of the two elements, such as by forming teams of participants from different age groups and establishing a competition mechanism to reward the best-performing team and most creative solution.

#### 4.3.2. Improve Communication and Empathy in Co-Creation

Effective co-creation between intergenerational groups can highlight individual values and facilitate communication [66]. In our study, the presence of a variety of topics and participants in the workshops necessitated adapting the rules and procedures in the organization phase. As such, we suggest that researchers should focus on establishing a communication system for different scenarios to deal with any problems that arise. A subdivision of the process (before, during, and after the workshop) may assist in this process. In our study, when selecting participants in the early stages of the workshop, we followed Hernandez et al. by distributing interest scales to ascertain which participants met the established criteria so that participants were on relatively equal footing [67].

To ensure that the participants understood the workshop content clearly, the toolkit was used to support the exchange of information during the workshop and to tailor the process to the characteristics of the community. The facilitator (designer) also played a mediating role by organizing interactive games to facilitate intergroup communication. After the workshop, the relationships between the participants were followed up and monitored so that the residents could report any changes in their perceptions, develop a positive understanding of individuals in other age groups, and build a sense of community through their participation in the workshop.

#### 4.3.3. Focus on the Process, Psychological, and Multidimensional Experiences in Group Co-Creation

Based on community psychology theory [68], multidimensional experience refers to the participants’ experience when they engage in co-creation, including the participants’ experience of the process and their feelings. In many cases, community members may feel frustrated by a lack of communication, to the extent that they refuse to participate in the activities, or by the contradiction between the theme culture and their worldview. These elements may prevent the event from running smoothly and may result in the event deviating from the intended goals. In addition, in the face of some publicly sensitive topics, such as intergenerational conflicts, it is important to pay attention to the rationality of the form and content of the interaction, so as to avoid reducing the group experience and causing the intensification of conflicts.

As such, the research team should plan the event in a holistic and detailed way. The planning process should involve establishing the theme in the early stages, finding ways to control the flow of the workshop, designing the toolkit format and materials in accordance with the details of the set-up, and organizing the space [69]. At the same time, it is important to focus on the cultivation of relationships and the breadth and depth of social interaction in response to audience and participant group diversity [70].

#### 4.3.4. Enhancing the Visibility and Effectiveness of Intergenerational Dialogue

The visibility of information communication can be understood as a form of expression that focuses on strengthening the dialogues between the various groups. Communication between audiences is not limited to verbal communication and therefore needs to be combined with other media channels [71]. For example, during the concept development phase of a workshop, the use of textual language is encouraged to develop open-ended topics to expand participants’ thinking. At the same time, the discussion and evaluation of the program should be presented graphically with data and information. These varying styles aid audiences in tracing the origin of the concepts during the discussion phase, which in turn allows audiences to quickly and accurately identify the concepts [72]. The visibility of co-creation methods aims to bring participants closer, offering the possibility for greater communicative and emotional experiences for all participants, including stakeholders [73,74].

#### 4.3.5. Utilizing the Natural Proactive Effectiveness of the Human Element

The types of intergenerational communication and interactions in different contexts in the community require targeted solutions through the values of the people themselves. As communities evolve, a new definition is given to community researchers: community-wide participatory planning that supports enablers. This involves community-wide planning, continuous participation in the whole chain of projects, and design facilitation, and it requires the researcher’s multi-dimensional capabilities such as coordination and resource integration.

Although the first axiom of the interaction perspective is that “a person cannot communicate” [75], in the process of conducting research and designing interventions, questions such as how to communicate with different groups of people, what form of interaction to use, and in what role to conduct research still need to be carefully considered. Combining previous theoretical foundations and design practice, we found that design workshops in community contexts make full use of the core advantages of intergenerational interaction and co-help, alleviating mutual stereotypes, promoting communication, obtaining a sense of identity, and enhancing well-being [69]. For example, when it comes to research on the rights and interests of the elderly in the community, it is possible to organize younger groups as the lead persons to communicate and interact with the elderly population, gradually building close and trusting relationships in a dynamic process, and then exploring the real needs of the residents. When organizing children’s groups for research practice, it is necessary to choose professional guides who are friendly and have a certain degree of prestige among the children so that the research can be carried out efficiently and orderly. In the case of multigenerational groups, it is necessary to use the resources of the group (residents, neighborhood committees, etc.) in multiple ways to mobilize the initiative of the group to participate. Therefore, it is important to choose the right researcher for the right activity topic to deal with the complex environment of the future community.

## 5. Conclusions

This study explored the possibility of using communication platforms and participatory activities to enable community residents to negotiate, cooperate, and articulate their real needs when public problems arise in a community. We found that despite differences and arguments in the process, positive-guidance-based intervention by a professional research team can resolve conflicts, reduce tension, and enhance residents’ enthusiasm and communication. Community groups of different ages facilitated intergenerational integration through mutual communication and collaboration in a cycle of collective activity. In addition, through our research practices, we concluded that the core of intergenerational integration lies in effective communication between intergenerational groups, equal collaboration, and strong empathy. This also shows that, through co-creation interventions, people are empowered to feel respected and gradually move from being served to being active contributors to the community [76]. We achieved this by incorporating the core conceptual thrusts of community psychology and also found that a collaborative co-creation approach effectively promoted intergenerational cognition, improved intergroup relations, and positively influenced community residents’ mental health.

Furthermore, our theoretical model combined the following strategies to promote intergenerational communication: (1) an integration strategy, which emphasizes the equality of relationships between groups; (2) a divergence strategy, which focuses on the individuality of groups and the abilities of each group; and (3) a synergy strategy, which builds a communication bridge between groups. Furthermore, to reduce negative mental health issues in intergenerational groups at the kernel level, we proposed strategies to shift the relationship from opposition to collaboration, improve both communication and empathy through co-creation, focus on the whole process experience by means of collaboration, and enhance the visuality and effectiveness of intergenerational dialogue.

However, this study has some limitations. First, public issues and conflicts in communities are complex and diverse, and this project was conducted in only one context. Moreover, although the intervention was designed to achieve significant results, it can only be used as a partial guideline. More research involving practical activities is needed to further explore the validity of the theory. In addition, the neighborhood in Shanghai, China, that was selected is not universally representative, and the higher civilizational literacy of its community residents aided in the recruitment of residents and the organization of activities. Therefore, more geographically and culturally diverse communities need to be examined in future studies.

In conclusion, we believe that the core of community building is to coordinate the relationships between people in the community environment. We also believe that it is necessary to abandon one’s original identity and gradually become a member of a community with a sincere heart, driven by the dynamics of time, and to adopt local cultural symbols. This study aimed to provide a possible reference for social innovation, community synergy, and future sustainable development by systematically studying and exploring the theory of intergenerational strategies. It is hoped that these results will be expanded and applied to other contexts to explore a wider range of developmental possibilities.

## Figures and Tables

**Table 1:** Intergenerational project plan.

Title	Program Steps	Implementation Content	Number of Days
Input Part	Step 1: Immersion observation to build a trusting relationship	We were looking for “community leaders” who can help us get to know the community as residents and participate in community square activities, observe the surrounding environment on their own, and get closer to the residents.	7
	Step 2: Community participatory research	Conduct targeted research activities based on community characteristics to understand the needs and problems of residents in general.	5
	Step 3: Community interview	Based on the specific needs of local residents for public space, one-on-one user interviews were conducted to further understand the core reasons behind the needs of the residents.	3
	Step 4: Establish a shared vision	Combined with preliminary team research and based on the specific needs of community residents, we invite community KOLs (opinion leaders) to participate in seminars and discuss the main issues that should be solved at the moment. For example, we will open a tea party conference to discuss the more sensitive conflicts in the public interest.	5
Output Part	Step 5: Collaborative workshop	Community KOL started by inviting residents of all age groups to conduct co-creation activities (workshops, etc.) on the topic.	7
	Step 6: Proof of concept and iteration	Based on the conceptual solutions co-created by the residents, suggestions are sought from more community residents and then iteratively updated.	5
	Step 7: Community illustration exhibition	The needs and views of community residents in public square space are demonstrated through community exhibitions.	7

**Table 2:** Community participant interview transcripts.

Interviewees	Interview Content
Aunt Wang (62)	“I appreciate what young people are doing for our community, listening to us carefully and giving positive and timely feedback to the community.”
Miss Song (36)	“My kids loved the ‘Truth or Dare’ event you held on the Community Day,” he said. “Two days ago, he was clamoring to take time off work to participate, saying that he played with many children in the square to answer the questions.”
Mr. Wu (10)	“At first I had a bad impression of the grandmothers in the square, but the last time I put stickers (co-created work parties) with them, they gave me a lot of advice and were really quite happy. It turns out that the grandmothers are in the square to protect us, so next time we should behave ourselves.”
Mr. Song (25)	“I was quite surprised, I did not expect the community to get so many activities, I was just in the community to pick up the courier or something. Then once I saw the square was very busy and took a look, it was quite interesting. Next time there are such activities I will call my friends to come together.”
Ant Gao (70)	“It was very touching to see the children in the square during our exhibition, and to think that we were really too aggressive with them and had to change our attitude. In the following days, there were children dancing with us and were actually very happy because they felt respected and recognized for what they were doing.”
Grandpa Li (72)	“You guys took note of this thing I mentioned about pulling up the height of the guardrail, and I’m very relieved I got it done quickly.”

**Table 3:** Intergenerational Attitudes Scale.

Age					SEX			
Please Fill in What You Think Teenagers/Young and Middle-Aged/Older People Are Like:								
	Strongly Agree	Agree	Somewhat Agree	Neutral	Somewhat Agree	Agree	Strongly Agree	
	7	6	5	4	3	2	1	
1. Pleasant								Good Complaint
2. Pleasant								Unpleasant
3. Friendly								Unfriendly
4. Intimate								Hostile
5. Kind-hearted								Bad-hearted
6. Tolerant								Intolerant
7. Cooperative								Uncooperative
8. Impartial								Unjust
9. Generous								Selfish
10. Thoughtful								Unresponsive
11. Patient								Impatient
12. Aggressive								Negative
13. Trusting								Suspicious
14. Reliable								Unreliable
15. Modest								Arrogant

## Data Availability

To protect the privacy of participants, the questionnaire data will not be disclosed to the public. If necessary, you can contact the corresponding author.
